# A Brief Targeted Review of Susceptibility Factors, Environmental Exposures, Asthma Incidence, and Recommendations for Future Asthma Incidence Research

**DOI:** 10.1289/ehp.8381

**Published:** 2006-01-26

**Authors:** Karin Yeatts, Peter Sly, Stephanie Shore, Scott Weiss, Fernando Martinez, Andrew Geller, Philip Bromberg, Paul Enright, Hillel Koren, David Weissman, MaryJane Selgrade

**Affiliations:** 1 Center for Environmental Medicine, Asthma, and Lung Biology, School of Medicine, University of North Carolina–Chapel Hill, Chapel Hill, North Carolina, USA; 2 Institute for Child Health, Division of Clinical Science, University of Western Australia, Perth, Australia; 3 Harvard School of Public Health and; 4 Harvard Medical School, Boston Massachusetts, USA; 5 University of Arizona, Arizona Respiratory Center, Tucson, Arizona, USA; 6 National Health and Environmental Effects Research Laboratory, U.S. Environmental Protection Agency, Research Triangle Park, North Carolina, USA; 7 University of Arizona, Tucson, Arizona, USA; 8 National Institute for Occupational Safety and Health, Morgantown, West Virgina, USA

**Keywords:** asthma, epidemiology, genetics, hygiene hypothesis, incidence, obesity, occupational asthma, smoking, susceptibility, windows of exposure and age (*in utero*, childhood, adult, elderly)

## Abstract

Relative to research on effects of environmental exposures on exacerbation of existing asthma, little research on incident asthma and environmental exposures has been conducted. However, this research is needed to better devise strategies for the prevention of asthma. The U.S. Environmental Protection Agency (EPA) and National Institute of Environmental Health Sciences held a conference in October 2004 to collaboratively discuss a future research agenda in this area. The first three articles in this mini-monograph summarize the discussion on potential putative environmental exposure; they include an overview of asthma and conclusions of the workshop participants with respect to public health actions that could currently be applied to the problem and research needs to better understand and control the induction and incidence of asthma, the potential role of indoor/outdoor air pollutants in the induction of asthma), and biologics in the induction of asthma. Susceptibility is a key concept in the U.S. EPA “Asthma Research Strategy” document and is associated with the U.S. EPA framework of protecting vulnerable populations from potentially harmful environmental exposures. Genetics, age, and lifestyle (obesity, diet) are major susceptibility factors in the induction of asthma and can interact with environmental exposures either synergistically or antagonistically. Therefore, in this fourth and last article we consider a number of “susceptibility factors” that potentially influence the asthmatic response to environmental exposures and propose a framework for developing research hypotheses regarding the effects of environmental exposures on asthma incidence and induction.

The documented increase in asthma prevalence over the last 25 years (Mannino et al. 2002) is likely due to changes in our environment or lifestyle because changes in our genetic makeup would take more than several generations to occur. When investigating environmental exposures [e.g., outdoor, environmental tobacco smoke, pollen, viruses (Gilmour et al. 2006; Zeldin et al. 2006)] in relation to asthma incidence and induction, we need to consider “susceptibility” factors in order to ascertain the relative contribution of an environmental effect and potential interactions. In this article, we define “susceptibility factors” broadly to include populations at risk (e.g., the very young, elderly, or genetically at risk), known risk factors, and known protective factors (“farm” exposure *in utero* or in infancy). From an epidemiologic framework, we have included variables that are sometimes considered confounders or effect modifiers, such as smoking or obesity (Figure 1).

Most epidemiologic research on asthma and environmental risk factors has focused on prevalence because prevalent asthma is easier to measure than incident asthma. Prevalence (*P*) is the product of the incidence (*I*) of the disease times the duration (*D*): *P* = *I* × *D*. Compared with prevalence, much less is known about factors associated with incident asthma. Thus, we include in this article factors associated with prevalent asthma, acknowledging that factors that influence the prevalence of the disease may not necessarily affect the incidence of the disease in the same way and that additional factors may affect the remission of the disease.

The following “susceptibility factors,” discussed at the U.S. Environmental Protection Agency (EPA) workshop “Environmental Influences on the Induction and Incidence of Asthma” held 19–20 October 2004, are considered in this article by experts in their fields: genetics, “window of exposure” (age: fetus, infant, childhood, adult, elderly), occupational asthma (OA), and lifestyle factors (diet, obesity). We define susceptibility as the degree to which a person or a population is sensitive to either adverse or protective exposures in developing asthma (U.S. EPA 2002). Smoking is also briefly mentioned here but is addressed in more detail in Gilmour et al. (2006). We also include a brief discussion of expanding the hygiene hypothesis. For population-level contextual analyses of susceptibility factors, disparities, and asthma, see Gold and Wright (2005), and for a thorough review of cohort studies on asthma incidence, see King et al. (2004).

## Genetics of Asthma and Gene–Environment Interactions

Studies of family history and genetic studies of familial aggregation and segregation of asthma have convincingly shown that the disease has a strong genetic component. Many investigators have sought to identify the specific genes and polymorphisms that determine hereditary susceptibility to the disease (Colhoun et al. 2003; Cookson 2004; Cookson and Moffatt 2004). A recent review of the literature identified more than 100 reports of genetic variants associated with asthma and asthma-related traits (Hoffjan et al. 2003). Among the more striking conclusions that can be reached from that review is that initial descriptions of “asthma” genes, identified both by linkage and association studies, have been difficult to replicate. No more than 8–10 such genes have been replicated in three or more studies, and none of these genes have been consistently associated with the same asthma phenotype in studies to date (Hoffjan et al. 2003).

Various technical reasons may explain these disappointing results. Type I and type II errors, for example, can result in spurious results or in results that cannot be replicated (Rothman and Greenland 1998) Similarily, publication bias (i.e., the underreporting of negative results) results in an exaggeration of the true statistical significance of any positive results (Rothman and Greenland 1998). Additionally, many replication studies either lack sufficient power or fail to define the phenotypes in ways that match the definitions used in the original work.

Although most of the literature on these issues has acknowledged these technical problems, analyses of the published results of population studies suggest that the discrepancies among results cannot be attributed only to deficiencies in study design or in the statistical analysis of the data. It is now clear that asthma is a heterogeneous condition (Douwes et al. 2002; Martinez 2002), that different forms of asthma may predominate in different geographic locations (e.g., the inner city or rural area), and that induction of asthma is strongly influenced by environmental factors that may differ among populations and at different ages (Eder et al. 2004, 2005). Moreover, different environmental exposures that increase or decrease the likelihood of developing asthma interact with different clusters of genetic variations. Almost paradoxically, then, a significant part of the genetic determination of asthma depends upon the environmental factors that trigger the disease (Patino and Martinez 2001).

Recently, for example, researchers in Germany, Austria, and Switzerland have shown that, in rural areas of those countries, children raised in the farm animal environment are much less likely to have asthma and allergic sensitization than are those raised in the same rural areas but away from such farms (Braunfahrlander et al. 2002). Furthermore, polymorphisms in the toll-like receptor 2 gene (*TLR2*; GenBank accession no. U88878; http://www.ncbi.nlm.gov) appear to modulate the protective effect of the farm environment: only carriers of certain *TLR2* polymorphisms are protected by this environment (Eder et al. 2004). Because *TLR2* is a receptor for microbial products (mainly gram-positive bacteria) that are known to be present in the farm animal environment, it is suspected (but not yet demonstrated) that these polymorphisms in *TLR2* may increase or decrease the reactivity of the individual to these products. Note that toll-like receptor 4 (*TLR4*; GenBank accession no. U88880; http://www.ncbi.nlm.gov) is the principal receptor for bacterial endotoxin, a glycolipid of the outer cell wall of gram-negative bacteria, whereas TLR2 is part of the signaling complex responding to cell membranes of gram-positive and -negative bacteria, mycoplasma, mycobacteria, parasites, and yeast (Eder et al. 2004; Lien et al. 1999). Similar gene–environment interactions have been described (Eder et al. 2005) for the potential association between allergic phenotypes and a polymorphism in the promoter region of the *CD14* gene (GenBank accession no. X06882; http://www.ncbi.nlm.gov). This gene encodes a receptor required for activation of *TLR4* by endotoxin. Thus, the identity of polymorphic genes modulating development of allergic sensitization may vary depending on specific environmental exposures. These findings further suggest that the development of specific T helper (T_H_) 2-type immunity (allergy) is strongly influenced by the state of activation of the innate immune system, the maturation state of the developing innate immune system at the time of microbial exposure, and the responsiveness of the latter to ligands that are generally derived from exogenous microbial products but may include endogenous compounds.

Genetic polymorphisms that influence susceptibility to the development of asthma exist, regardless of the environment in which the individual is raised or lives, and similarly, environmental exposures, may influence to some degree the susceptibility to allergic airways disease in the absence of genetic predisposition (Eder et al. 2005). However, it is likely that the risk of developing asthma is greatest when both genetic and environmental risk factors are present simultaneously. Evidence to date suggests that asthma induction is the result of complex interactions between specific exposures and the genes that interact with such exposures during crucial periods in the natural history of the disease. The current “epidemic” of asthma cannot be explained by genetic factors, but genetic factors may still be important with regard to susceptibility issues.

## Windows of Exposure

### *Influence of* in utero *and postnatal events.*

The *in utero* environment is the first environment to which an individual is exposed. Exposures *in utero* occurring during the critical periods for organogenesis have the potential to produce long-lasting effects. Postnatal organ function can be profoundly affected by *in utero* exposures (Stick et al. 1996), and the “fetal origins of adult disease” have attracted considerable research attention recently. Postnatal respiratory function is largely determined by lung development *in utero*, with strong tracking of respiratory function throughout childhood (Hibbert et al. 1990). Specifically, children born with low lung function are likely to have low lung function throughout childhood and into adult life.

A number of other factors must also be considered when assessing the effects of *in utero* exposures on the risk of developing asthma (Table 1). Also important are timing of T-cell memory programming, genetic predispositions for allergy and infections, and synergy of early respiratory infection and atopy.

Children born to atopic parents are at increased risk of developing allergic sensitization to common aeroallergens and asthma (Holt et al. 1999). The child’s adaptive immune system is functionally immature at birth and undergoes slow postnatal maturation (Yabuhara et al. 1997). Although genetic factors may influence immunologic development, environmental influences are also possible. For example, cord blood IgE levels are highest in first-born infants, and the *in vitro* proliferation response of cord blood mononuclear cell (CBMC) to mitogenic stimuli decreases with birth order. A plausible explanation for this phenomenon is an increasing response of the maternal immune system to “paternal” human leukocyte antigens in fetal cells. Such a response is likely to be of the T_H_1 type and is likely to counterbalance the T_H_2 skewed fetal and neonatal responses. Pregnancy complications (McKeevar et al. 2002; Stazi et al. 2002) and delivery by cesarean section are risk factors for asthma and allergies (Negele et al. 2004). There is a synergistic interaction between infections in early life and development of atopy and subsequent asthma by 6 years of age (Oddy et al. 1999).

The fetal and maternal placental tissues secrete a variety of cytokines, including “acute-phase” cytokines [interleukin (IL)-6, IL-10] and “anti-T_H_1” cytokines [IL-4, IL-10, IL-13, tumor growth factor 2 (TGF2)]. In addition to being essential for continued pregnancy, these cytokines may influence the developing fetal immune system and be responsible for the T_H_2 bias seen in stimulated CBMCs *in vitro*. Infants born to smoking mothers have lower levels of cytokines (both T_H_1 and T_H_2) in their cord blood, demonstrating that placental trophoblasts are vulnerable to *in utero* exposures (Macaubas et al. 2003), which could result in immune dysregulation.

The question of whether fetal priming to environmental allergens occurs *in utero* is controversial. It is not certain whether allergens cross the placenta *in vivo*; although this has been demonstrated using perfused placental models *in vitro*, the relevance of these findings is unclear. The results of strict dietary allergen-avoidance strategies during pregnancy (intended to decrease the severity of allergy and asthma in the offspring) have been disappointing and may even increase the severity of clinical atopy when exposure occurs postnatally (Kramer and Kakuma 2003). CBMC responses to aeroallergens have been demonstrated using *in vitro* stimulation protocols. However, several lines of evidence suggest that these responses should not be taken as evidence for *in utero* priming to allergens (Thornton et al. 2004). This evidence includes the lack of both a qualitative and a quantitative difference in responses from CBMC obtained from high-risk and low-risk infants (Prescott et al. 1998, 1999) and the fact that CBMC responses to seasonal allergens are not restricted to mothers exposed to the allergen during pregnancy.

The influence of maternal diet on infant atopy outcomes is unclear. Maternal diets high in linoleic acid have been associated with increased cord blood IgE levels and increased asthma. Fish oil supplementation has been associated with lower CBMC cytokine responses to *in vitro* stimulation in one study (Dunstan 2003). Maternal diets high in antioxidants have been associated with decreased CBMC proliferative responses, which may not necessarily indicate a beneficial effect, but also with decreased wheeze and atopic dermatitis during infancy. The data on the effects of fetal nutrition (both over- and undernutrition) are contradictory, both between and within studies (Katz et al. 2003; Kramer and Kakuma 2003).

Maternal smoking during pregnancy is a major *in utero* exposure that is associated with poor fetal and infant outcome and increased wheezing and asthma in children (Stick et al. 1996). The risk of developing asthma among children 7 years of age increased in a dose-dependent pattern with the mother’s smoking rate in pregnancy: odds ratios were 1.25 (95% confidence interval (CI), 1.09–1.44) for < 10 cigarettes/day and 1.36 (1.14–1.63) for > 10 cigarettes/day (Jaakkola and Gissler 2004) in a Finnish birth cohort that included almost 60,000 children. Significant associations persisted even after adjustment for low birth weight and duration of pregnancy, suggesting that the effect of passive smoking on asthma was independent of these factors.

The major harmful components of cigarette smoke are nicotine and carbon monoxide (Longo 1977; Sekhon et al. 2001). The physiologic consequences of exposure include constriction of uteroplacental circulation, increased release of catecholamines, and decreased fetal breathing movements. The adverse outcomes include decreased birth weight, decreased cord blood cytokines levels (both T_H_1 and T_H_2), increased collagen deposition in airway and alveolar walls, abnormal lung function at birth, and suppressed ventilatory responses to postnatal hypoxia (Sekhon et al. 2002; Ueda et al. 1999). Prenatal alcohol and nicotine exposure have additive effects on suppressing peripheral blood mononuclear cells proliferative responses in the early postnatal months (Basta et al. 2000).

In summary, *in utero* exposures can play an important role in the development of atopy and asthma. Maternal smoking is a major preventable risk factor for asthma, and fetal nutrition and the influence of maternal diet (notably antioxidants and omega-3 fatty acids) are also important. The data on the effects of other environmental agents are still equivocal. CBMC studies need to be interpreted cautiously, and evidence for *in utero* priming remains dubious. Table 1 summarizes these and other environmental factors.

### Childhood.

The following paragraphs include descriptions of prevalent rather than incident asthma because little is known about incident childhood asthma. Asthma is the most common chronic childhood disease (Yunginger et al. 1992). In addition, there is substantial wheezing in the general population in early life such that 40% of all children have a sustained wheezing illness in the first year of life and 20% are wheezing at 3 years of age, with a similar percentage at 6 years of age (Martinez et al. 1995). Considering observations that the mean duration of symptoms in childhood is 2 years, it is clear that recall bias for early-life symptoms is considerable (Sears et al. 2003). Thus, recrudescence of symptoms is common, and it is difficult, in adults, to determine if the disease is truly incident or if it is the reoccurrence of symptoms in a genetically susceptible individual.

Estimates of the effects of recall bias can be made from prospective epidemiologic studies. From 30 to 50% of children will forget that they had the diagnosis of asthma or wheeze symptoms 3 years earlier (Sears et al. 2003). A major challenge is that we are still unable to predict who will have their wheeze symptoms persist and who will remit, based on their presentation in early life.

There are several factors that are predictive of asthma in childhood. Parental history is an index of genetic susceptibility to asthma: 80% of children with two asthmatic parents develop the disease compared with 40% of children with one asthmatic parent and 10% of children with no asthmatic parents (Sibbald et al. 1980). Sex is also a powerful predictor. Males develop the disease 2 to 4 times more frequently than females in the first 3 years of life, but females are more likely to have the disease persist, especially if they have it after puberty (Yunginger et al. 1992). This results in equal prevalence of the disease by sex after 10 years of age, but females are more likely to die and/or be hospitalized than males after this age. Other predictors include the degree of airway reactivity and the presence of other atopic diseases such as atopic dermatitis, allergic rhinitis, food allergy, and urticaria. The more severe the symptoms, the more likely the disease will persist.

### Young adulthood.

Relatively little is known about the important predictors of asthma in young adulthood. Recently Wang et al. (2004) reported on the importance of symptoms, airway responsiveness, eosinophilia, and smoking as factors reducing maximally attained levels of lung function in this age group. Smoking interacts with airway responsiveness and is associated with a higher likelihood of new onset (or recrudescent) asthma in adulthood. Asthmatics with more severe disease are more likely to have been smokers.

### Working-adult occupational asthma.

Work place exposures are responsible for 15–25% of asthma in adults (Arif et al. 2003a, 2003b; Balmes et al. 2003; Blanc et al. 2003; Karjalainen et al. 2001). Hence, most information on factors affecting adult onset asthma comes from occupational studies. New-onset work-related asthma can be subcategorized into OA and irritant-induced asthma. OA specifically refers to asthma that occurs as a consequence of sensitization to causative agents in the workplace, usually after an exposure history of months to years (Meijer et al. 2004). Sensitizing agents that cause OA can be divided into high-molecular-weight (HMW) and low-molecular-weight (LMW) agents (Chan-Yeung et al. 2003). In cross-sectional studies, atopy has been a well-documented risk factor for IgE sensitization to occupational HMW allergens. At least one cohort study suggests that atopic phenotype can also be an outcome of exposure to HMW occupational allergen (Nguyen et al. 2003). In general, HMW (≥5 kDa) agents are proteins and cause OA via IgE sensitization. In contrast, LMW (< 5 kDa) agents are considered too small to induce immune sensitization by themselves. They are thought to act as haptens, binding to carrier proteins to form complete antigens with the ability to induce immune responses. Although some LMW agents such as platinum clearly induce OA via IgE sensitization, this is less clear for others such as isocyanates and plicatic acid (Weissman and Lewis 2000). Effector mechanisms other than those mediated by IgE, such as T-cell responses, probably have important etiologic roles in OA induced by these agents, and atopy is not a risk factor.

OA has been successfully prevented in several settings (Cullinan et al. 2003). An impressive example was elimination of asthma outbreaks in the enzyme detergent industry in the late 1960s and early 1970s (Cathcart et al. 1997; Schweigert et al. 2000). A more recent example has been marked reduction in OA caused by powdered natural rubber latex gloves in the health care industry (Allmers et al. 2002; Liss and Tarlo 2001). Guidelines have recently been provided by the American Thoracic Society (2004) on managing asthma risk at work, school, and recreation that are applicable to a range of asthma-inducing agents.

However, cohort studies that objectively assess exposure–response relationships for causative agents are available for only a minority of agents. Furthermore, studies have generally focused on inhalation exposure and have not attempted to address the roles of other potential routes of exposure such as skin, upper respiratory tract, and mucus membranes in development of sensitization and disease (Weissman and Lewis 2002). In some situations such as asthma attributed to poor indoor air quality, causative agents remain unclear, although Park et al. (2004) has indicated that semiquantitative indices of exposure to dampness and mold can suffice for measuring the effect of poor workplace indoor air quality on respiratory symptoms. Cox-Ganser et al. (2005) show that occupancy of water-damaged buildings by workers was associated with onset and exacerbation of respiratory conditions, confirmed by objective medical tests. Bornehag et al. (2004) also provide a comprehensive review on dampness and mite work-place exposure in buildings and their associated health effects. Indoor air exposures and incident asthma are discussed in more depth by Gilmour et al. (2006), Zeldin et al. (2006), and the Institute of Medicine (2000, 2004). Depending on the work setting and causative agent, conflicting data have been published about the role of co-exposures such as tobacco smoke (Gilmour et al. (2006). Finally, as is true for common asthma, important questions remain about genetic and other individual susceptibility factors.

### Older age.

On a national level the overall prevalence of self-reported asthma in the United States increased from 3.2 to 4.6% in people 65 or more years of age between 1980 and 1996 (Mannino et al. 2002). As the total number of people older than 60 years increases [it is predicted to double between 2000 and 2059 (Kalache and Keller 2000)], the number of elderly people with asthma will increase. Moreover, asthma is underdiagnosed and poorly treated in older adults and substantially reduces quality of life (Bellia et al. 2003; Dow et al. 2001; Enright et al. 1999; Huss et al. 2001; Parameswaran et al. 1998). The prevalence of asthma in an older population sample of four U.S. communities was 4% for physician-diagnosed asthma and another 4% for probable asthma (symptoms of asthma during the previous 12 months but no diagnosis or treatment) (Enright et al. 1999). Older adults report the onset of their asthma relatively equally over all the decades of life (Enright et al. 1994).

Exposures associated with asthma in older adults include indoor and outdoor air pollution, occupational exposures, and active and passive smoking. Allergen sensitization has been associated with asthma in older adults (Litonjua et al. 1997; Weiss et al. 1998). Older adults have smaller airways compared with young adults, and this makes bronchial hyperresponsiveness more likely (Britton et al. 1994; Peat et al. 1992).

The Centers for Disease Control and Prevention has yet to conduct longitudinal studies of asthma incidence. Although the increased prevalence suggests that the incidence has also risen, based on currently available data, it is hard to know how much of the asthma seen in older adults is newly developed disease and how much is persistence or recurrence or exacerbation of disease developed earlier in life.

Up to half of older adults with asthma are current or former smokers (Enright et al. 1999). Smokers with airway hyperreactivity are more likely to experience faster decline in forced expiratory volume in 1 sec than smokers without airway hyperreactivity. Cigarette smoking enhances the production of IgE antibodies, stimulates the production of inflammatory markers in the sputum, and causes hyperinflation in older asthmatics (Mitsunobu et al. 2004). In Sweden, adult-onset asthma was associated with exposure to molds, environmental tobacco smoke (ETS), and the presence of a wood-burning stove (Thorn et al. 2001). In Finland, indoor dampness and mold growth was found to contribute to adult-onset asthma (Jaakkola et al. 2002).

Asthma in older women living in Northern California was associated with occupations in art, decorating, photography, technology, health professions, food preparation, cleaning, social work, and service work (Forastiere et al. 1998), as well as with smoking and exposures to dusts, gas, vapors, fumes, or sensitizers. Asthma in older women in developing countries such as India is associated with the use of biomass fuels for cooking or home heating, especially when the stove and sleeping quarters are located in the same room (Mishra 2003).

If the asthma phenotype is more heterogeneous in older adults compared with children, the strategies needed to prevent new-onset cases, as well as exacerbation of existing asthma, in this population may be different from those needed to protect children. To develop effective strategies, we need studies that specifically target older adults.

## Lifestyle: Obesity

Obesity is a large and rapidly growing public health problem both in the United States and worldwide. From 1976 through 1994, the prevalence of overweight children and adolescents in the United States almost doubled (Jackson 2003). Numerous cross-sectional epidemiologic studies indicate an increased prevalence of asthma in the obese (Arif et al. 2003a; Chinn 2003; Luder et al. 2004; Schachter 2003).

Obesity may be particularly important for severe asthma because a large epidemiologic survey indicates that > 75% of subjects visiting emergency departments for asthma are obese or overweight (Thomson et al. 2003). These asthmatics had severities of airflow obstruction similar to those of lean subjects visiting emergency departments for asthma and responded equally well to standard asthma therapy, indicating that asthma in obese individuals does not simply reflect misdiagnosed dyspnea.

It is important that we understand the mechanistic basis for the relationship between obesity and asthma, both because the prevalence of obesity is extremely high among inner-city children (Luder et al. 1998), who have a particularly high prevalence of asthma, and because obesity is a strong predictor of the persistence of childhood asthma into adolescence (Guerra et al. 2004). The relationship is much stronger in females than in males. In a birth cohort study of 1,000 individuals (Hancox et al. 2004), raised body mass index (BMI) was associated with asthma and atopy in women but not in men. Population attributable fraction calculations estimated that 28% (95% CI, 7–45) of asthma developing in women after 9 years of age is due to being overweight (Hancox et al. 2004).

Longitudinal studies controlling for other confounding variables, including exercise, indicate that obesity antedates asthma and that the relative risk of incident asthma increases with increasing obesity (Camargo et al. 1999; Castro-Rodriguez et al. 2001; Gold et al. 2003; Guerra et al. 2004). Furthermore, morbidly obese asthmatics studied after weight loss demonstrate decreased severity and symptoms of asthma (Aaron et al. 2004; Stenius-Aarniala et al. 2000). It is likely, therefore, that obesity somehow either causes or exacerbates asthma.

The mechanistic relationship between obesity and asthma has been studied using mouse models of the disease. *Ob/ob* mice, which are genetically deficient in the satiety hormone leptin [leptin; GenBank accession no. U22421, mouse (*Mus musculus*); http://www.ncbi.nlm.gov]; *db/db* mice, which lack the leptin receptor isoform 1 gene [*lepr*; GenBank accession no. AF039443 (mouse); (http://www.ncbi.nlm.gov)]; and Cpe^fat^ mice, which are genetically deficient in an enzyme involved in cleavage of neuropeptides involved in satiety, are all obese. Each of these mouse strains demonstrates innate airway hyperresponsiveness that is not the result of differences in absolute lung volume or tidal volume. It has been hypothesized that this airway hyperresponsiveness may be related to chronic low-grade systemic inflammation, a characteristic of both human and murine obesity (Bullo et al. 2003; Takahashi et al. 2003).

Compared with lean mice, obese mice also demonstrate enhanced increases in airway inflammation and airway responsiveness after exposure to ozone, a common asthma trigger (Rivera-Sanchez et al. 2003; Shore et al. 2003). After allergen sensitization and challenge, obese mice also have increased changes in baseline pulmonary mechanics, airway responsiveness, and serum IgE compared with lean mice. The relationship between obesity and atopy has been less well studied in humans than the relationship between obesity and asthma, but there are some epidemiologic studies indicating that higher BMI is associated with increased prevalence of atopy (Huang et al. 1999; Schachter et al. 2003).

Increases in serum leptin observed in obesity may contribute to the increased incidence of asthma in the obese because leptin, a member of the IL-6 family of cytokines, has pro-inflammatory effects. Sensitized wild-type mice challenged with ovalbumin developed increased serum leptin, consistent with a recent report that for comparable BMI, leptin is increased in asthmatic compared with healthy boys (Guler et al. 2004). Exogenous leptin administration in sensitized mice increased allergen-induced increases in airway responsiveness and serum IgE without any effects on airway inflammation or T_H_2 cytokine expression (Shore et al. 2005). The results suggest that leptin has the potential to exacerbate asthma in the obese.

Other aspects of lifestyle that are not discussed in depth here include diet and exercise separate from their relationship to obesity, breast-feeding, and “healthy” lifestyles. For example, there may be a role for antioxidants, omega-3 fatty acids, nonrefined carbohydrates, or other nutrients in the prevention of asthma, although to date the evidence is not conclusive.

## The “Hygiene Hypothesis”

In the late 1980s, as the results of the air pollution and respiratory infections studies on asthma were being assimilated by the scientific community, a critical observation was reported in the literature noting a strong inverse relationship between household size and hay fever in a cohort in the United Kingdom (Strachan 1989). Contrary to the prevailing paradigm that respiratory infection induces asthma, Strachan postulated that the association of large household size and less hay fever could be explained if allergic diseases were prevented by infection in early childhood, transmitted by contact with older siblings. This theory was colloquially termed the “hygiene hypothesis.”

In support of this hypothesis, historical epidemiologic evidence suggests that although the incidence of infectious disease in childhood decreased, allergic disease emerged as a post-industrial revolution phenomenon during the 19th century and continued to increase in prevalence throughout the 20th century (Strachan 2000). Childhood infectious diseases were therefore hypothesized to have a protective effect on the subsequent development of allergic disease.

In global collaborative research, international comparisons such as the International Study of Asthma and Allergies in Childhood (ISAAC) found a 15-fold variation in prevalence of asthma symptoms among countries. Economically developed countries tended to have the higher prevalence (with English-speaking countries having the highest) (ISAAC Steering Committee 1998). Asthma prevalence was also lower in preunification East Germany than in West Germany (von Mutius et al. 1994). These epidemiologic data are compatible with the hygiene hypothesis.

Other epidemiologic evidence found to support the hygiene hypothesis include a higher prevalence of eczema, skin prick positivity, and allergen specific IgE in children brought up in smaller and more affluent families and in first-born children (Strachan 1989; Wickens et al. 1999). Early exposure to childhood infections due to daycare attendance at young ages was also linked with a lower incidence of asthma in a Tucson, Arizona, cohort (Ball et al. 2000).

More recently, Braunfahrlander et al. (2002) and von Mutius et al. (2000) showed a reduced risk of hay fever, asthma, and atopy in rural-dwelling children from farming families compared with nonfarming rural families. These studies have consistently suggested that protective exposures for asthma and allergies are present in the stables and barns of farm animals. Endotoxin, a component of the cell walls of gram-negative bacteria (which is ubiquitous in the modern environment but reaches much higher levels in areas where livestock animals are housed), has become a main environmental exposure of interest. Mechanistically, it is thought that exposure to microbial products, and endotoxin specifically, stimulates the young immune system to develop the T_H_1 response pathways instead of the T_H_2 path-ways and thereby protects against the development of asthma and allergy. Other supportive evidence on factors that may program the initial susceptibility to asthma and allergy include studies of parasitic infections (Yazdanbakhsh and Wahyuni 2005), studies on the use of acetaminophen/paracetamol in pregnancy (Shaheen et al. 2005), and migrant studies (Gold and Acevedo-Garcia 2005).

However, the hygiene hypothesis assumes that once the immune system profile is developed in the infant or child, there is no turning back. Yet there is evidence that the immune system is not “fixed” after the first years of life; that is, “immune deviation” may take place throughout life, as OA illustrates (Douwes et al. 2004). For example, Portengen et al. (2005) report that endotoxin exposure in adult pig farmers protects against allergic sensitization.

On the other hand, some risk factors for asthma do not neatly fit into the hygiene hypothesis. These include ETS exposure (particularly if a mother smokes in pregnancy), respiratory syntactical virus (RSV) infection, obesity, pesticide exposures (Hoppin et al. 2003), OA (Elliot et al. 2005), air pollution (McConnell et al. 2002), and living in inner-city communities, which have high prevalences of asthma across the country. King et al. (2004) present a thorough review of studies of factors that have been associated with asthma incidence to date.

Holt et al. (2005) propose that there may be interactions between airway tissue damage in early life caused by viral infections and inhalant allergy in asthma etiology. We propose a hypothesis that could account for both the hygiene hypotheses and other risk factors, that is, synergistic and/or antagonistic interactions between immune function development or change (at later stages of life) and structural damage to the lung (via air pollution, ETS, RSV) occurring at different time points throughout life.

The challenge for the future is to identify the factors that confer the protection proposed by the hygiene hypothesis and to find strategies to modify the environment to reduce the incidence of asthma and T_H_2 sensitization to common environmental antigens without causing harm to susceptible individuals.

## Additional Thoughts on Asthma Incidence and Induction Research

Much of the ongoing asthma research is based on “high-risk” cohorts that maximize inclusion of children with a family history of asthma and recruit primarily children with allergic asthma. The knowledge gained from these studies, although important and useful for high-risk children, does not represent the entire population. There seems to be an assumption that the only good knowledge left to be gained epidemiologically will come from future and existing cohort studies. Yet, case–control studies and cross-sectional studies are still quite useful and much less costly, take much less time, and can be used for hypothesis generation. The studies by Braunfahrlander, von Mutius, and colleagues, described previously, are a case in point. An astute observation that farm children have a lower prevalence of asthma than non-farm children has spawned an entire field of research into endotoxin and toll-like receptors.

There is a dearth of research into factors associated with asthma and allergy remission. Remission studies can be highly informative about the natural history of the disease. In one of the few reports in this area, Sears et al. (2003), in a population-based (rather than high-risk) cohort of children, showed that the later the age of disease onset in childhood, the less likely the child would relapse later in adulthood. It also demonstrated that smoking was strongly associated with persistent asthma.

Animal models have been developed primarily for the study of allergic sensitization rather than asthma. Perhaps toxicologists and immunologists can also broaden their efforts to improve animal models of asthma development. Collaborative research among epidemiologists, toxicologists, immunologists, allergists, occupational hygienists, and pulmonologists will help us reach our goal of developing strategies to prevent asthma in future generations.

## Conclusion

There is substantial evidence that genetics, obesity, and the window of exposure (fetus, infant, elderly) are major susceptibility factors in the pathogenesis of asthmatic disease. Future research on the incidence and induction of asthma should take susceptibility factors into account. New research findings should then be used to generate public health interventions aimed at preventing a further rise in asthma and ultimately to reverse the existing trend.

## Figures and Tables

**Figure 1:**
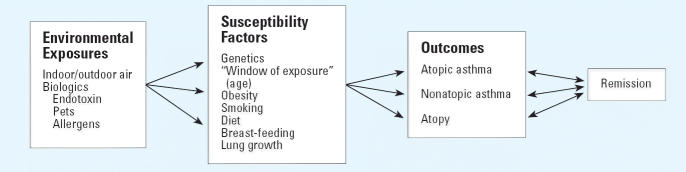
Conceptual framework for susceptibility factors.

**Table 1:** Summary of possible antenatal influences on the development of asthma and allergy.

	Allergy	Asthma
Birth order	+	+
Maternal allergen exposure	+/−	−
Maternal smoking during pregnancy	+/−	++
Obstetric complications	+	+
Elective cesarean section	−	+
Maternal use of antibiotics during pregnancy	+/−	+/−
Maternal diet, PUFA, antioxidants	+/−	+/−

Abbreviations: PUFA, polyunsaturated fatty acids; +, positive association with disease outcome; −, negative association with the disease outcome; +/−, equivocal data.
